# Spatial and temporal heterogeneities of district-level typhoid morbidities in Ghana: A requisite insight for informed public health response

**DOI:** 10.1371/journal.pone.0208006

**Published:** 2018-11-29

**Authors:** Frank Badu Osei, Alfred Stein, Sylvester Dodzi Nyadanu

**Affiliations:** 1 Faculty of Geo-Information Science and Earth Observation (ITC), University of Twente, Enschede, Netherlands; 2 ECHO Research Group International, Aflao, Ghana; University of Miami, UNITED STATES

## Abstract

Typhoid fever is estimated to cause between 9.9–24.2 million cases and 75,000–208,000 deaths per year globally. Low-income and middle-income countries report the majority of cases, especially those in sub-Saharan Africa. The epidemiology of typhoid fever is poorly understood, particularly in Ghana where there has been no study of the within-country variation. Our objective was to explore and analyze the spatial and temporal patterns of typhoid fever morbidities in Ghana. We used the global and local Moran’s indices to uncover the existence of global and local spatial patterns, respectively. Generalized linear autoregressive moving average (*glarma*) models were developed to explore the overall and regional level temporal patterns of morbidities. The overall index of spatial association was 0.19 (*p* < 0.001). The global Moran’s monthly indices of clustering ranged from ≈ 0 − 0.28, with few non-significant (*p* > 0.05) estimates. The yearly estimates were all significant (*p* < 0.001) and ranged from 0.1–0.19, suggesting spatial clustering of typhoid. The local Moran’s maps indicated isolated high contributions of clustering within the Upper West and Western regions. The overall and regional level *glarma* models indicated significant first and second-order serial correlation as well as quarterly trends. These findings can provide relevant epidemiological insight into the spatial and temporal patterns of typhoid epidemiology and useful to complement the development of control strategies by public health managers.

## Introduction

Typhoid is a bacterial enteric infection caused by *Salmonella enterica serovar Typhi* and *Salmonella enterica serovar Paratyphi*. Transmission is directly through contact with faecally contaminated water or food [1,2]. This is the most common cause of enteric fever and is estimated to cause between 9.9–24.2 million cases and 75,000–208,000 deaths per year globally [3–5]. The burden of typhoid fever is greater for lower and middle-income countries (LMICs), especially in Asia and sub-Saharan Africa, and in parts of the Middle East and Latin America.

In sub-Saharan Africa, available data to explain the epidemiology is limited. Recent studies have focused on regional level multicenter population-based surveillance to estimate the burden [5,6]. Mogasale et al. [5] estimated the burden in LMICs, after adjusting for water-related risk, to be 11.9 million cases and 129,000 deaths. Using a model-based approach, Antillón et al [7] estimated the burden in LMICs to be 17.8 million, ≈ 50% higher than the estimates of Mogasale and colleagues [5]. These estimates, however, were based on regional and national level data, without consideration for within-country variations. Knowledge of the spatial and temporal patterns of the disease burden is crucial for scaling up informed public health strategies, allocation of resources, and monitoring of interventions [8].

In Ghana, typhoid fever ranks amongst the leading causes of outpatient illnesses. Incidence was estimated to be 120 per 100,000 people among children between 0 and 15 years [9]. Epidemiological studies of typhoid in Ghana are rare; our literature search found only two studies that estimated the systematic bacteremia infection among children in a rural area in the Ashanti Region [9,10]. There is an unquestionable paucity of knowledge on the within-country variation of the burden, hence, limits the ability of health policymakers to evaluate and implement appropriate preventive measures.

Mapping the spatial distribution, identification of the spatial patterns and evaluation of the temporal dynamics of diseases has become indispensable in public health. In this study, our objective is to evaluate the spatial patterns and model the heterogeneities of the temporal dynamics of typhoid fever in Ghana, dwelling on district level morbidity data. The role of key indicators like poverty and socioeconomic circumstances, and climate-related factors [4,7] is a consequential indication of the existence of spatial clustering and temporal heterogeneity in Typhoid incidences. Espinoza et al [11] have observed differences in *Salmonella* incidences between rural and urban children in Ghana. This is suggestive that typhoid risk is uneven or spatially varied, the nature of which, either clustered or random, is yet unknown. Previous studies [12,13], which suggest that typhoid fever occurs predominantly in urban settlements with high population densities support this argument. Currently, until access to safe drinking water and improved sanitation is expanded, vaccination presents an unprecedented opportunity, especially in endemic settings [14,15]. But there are still questions of where to prioritize in order to optimize scarce resources. Therefore, evaluating and understanding within-country or small-area spatial variation and temporal dynamics is of particular salience to the design and implementation of optimal vaccination strategies.

Spatial statistical methods such as the global and local Moran’s indices (Moran’s *I* hereafter), developed for continuous data or Gaussian data [16–18], can uncover the existence of spatial patterns of disease rates and offer quantitative indications to compare where these patterns are relatively high or low. Transformation of disease counts to rates may yield approximately Gaussian distribution. However, the raw rates could be over-dispersed with unstable variances due to spatial autocorrelation and population differentials. Empirical Bayesian (EB) smoothing and standardization can account for the unstable variances arising from populations differentials [19], while the conditional autoregressive (CAR) model can account for over-dispersion due to spatial autocorrelation [20]. The generalized linear autoregressive moving average (*glarma*) models are observation-driven class of models with a robust way to detect and account for serial dependence in times series of counts [21]. In this study, we use both the global and local Moran’s *I* to quantify spatial autocorrelation of EB standardized rates, and the EB smoothing and CAR model to map the spatial distribution of typhoid rates. We further develop *glarma* models to evaluate and explore the temporal dynamics of typhoid occurrences.

## Materials and methods

### Study area and data

**Fig 1:**
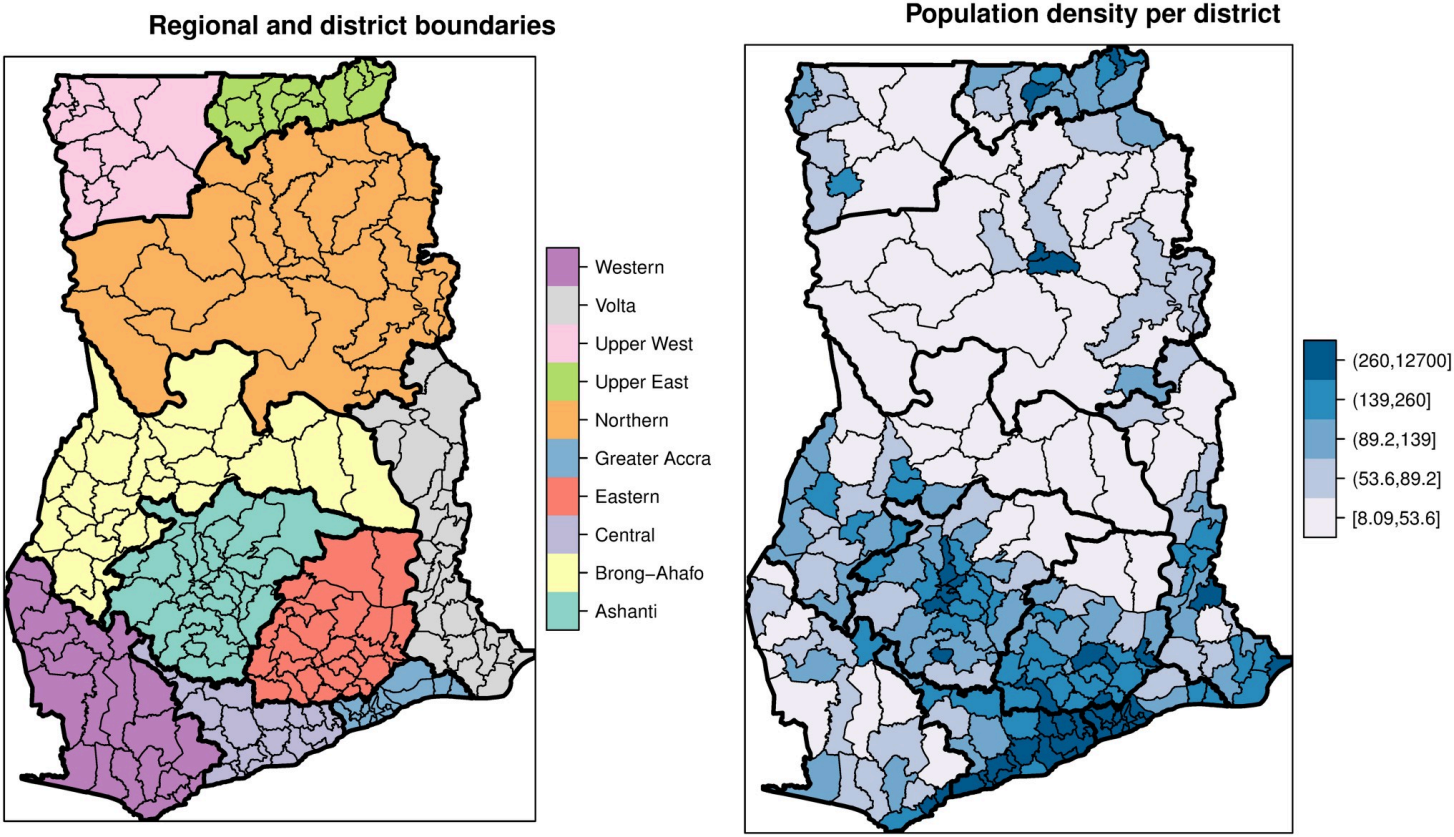
Boundary (district and regional) and population density maps. These maps show the district (Faint lines) and regional (Bold lines) boundaries and the spatial distributions of population densities (persons per kilometers square).

Ghana is centrally located on the west coast of Africa with a total land area of 238,589 km^2^. It is a tropical region that is strongly influenced by the West African Monsoon, hence, with varying temperatures and rainfall intensities. Ghana undergoes variations in climate that are sometimes marked by severe droughts and floods. The country consists of ten administrative regions, subdivided into 216 districts (Fig 1). Projections by the Ghana Statistical Service (GSS) puts the 2017 population at 27,043,093. Population data per district were obtained from the Ghana Statistical Service (GSS). The population density (people per square kilometers) vary widely across districts (Fig 1) and heavily skewed to the right, with the mean being 468.3 and standard deviation 1564.5 In Ghana, reporting facilities (health centers, clinics, and hospitals) report all morbidities through a District Health Information Management System (DHIMS). Within each district, there is at least one reporting facility. The data are then managed by the Centre for Health Information and Management (CHIM) of the Ghana Health Services (GHS). In this study, we obtained the monthly typhoid morbidities, from January 2011 to December 2015, for the 216 districts from the CHIM.

### Mapping the spatial distribution

Consider the typhoid incidences *y*_*it*_, *i* = 1,…, *m* = 216 districts and *t* = 1,…, *T* = 60 months (Jan 2011 to December 2015) as being independently Poisson distributed *y*_*it*_~*Poi*(*n*_*it*_*r*_*it*_) with mean proportional to the unknown risk *r*_*it*_, where *n*_*it*_ is the number of persons at risk in district *i* at year *t*. The maximum likelihood estimates of the risk or the standard morbidity ratio (SMR) is ${\hat{r}}_{it}=y_{it}/n_{it}$. Mapping the crude risks has drawbacks. Firstly, the crude risks have variances inversely proportional to their populations. Hence, sparsely populated districts may spuriously suggest high rates when mapped. To overcome this, we used the local EB smoothing to estimate variance-stabilized estimates of the crude risk. This smoothing consists of obtaining a weighted average between the raw estimates for each district and the neighboring average, with weights proportional to the underlying population at risk [22,23] (see S1 File for details). Secondly, the crude risks are estimated under the assumption of independence between geographical areas, an assumption unrealistic for infectious diseases due to common risk factors for adjacent districts. Consequently, the crude risk estimates do not account for this over-dispersion. Here, we used the CAR [20] to account for over-dispersion due to spatial autocorrelation. The CAR model relates the risk at a specific district *i* to that of its immediate neighbours *J*_*i*_, specified by means of a spatial neighborhood structure *w*_*ij*_. We defined the spatial neighborhood structure *w*_*ij*_ as a binary connectivity weight matrix; *w*_*ij*_ = 1 if districts *i* and *j* are adjacent, and 0 otherwise. Detailed description of the CAR model and its applications can be found in [20,24,25].

### Exploring the spatial patterns

We used the global Moran’s *I* [16] to estimate the overall strength of spatial autocorrelation and its local equivalent, Local Indicator for Spatial Association (LISA) [17], to estimate the spatial autocorrelation between districts and their neighboring districts. We applied the EB standardization on order to account for the unequal variances arising from unequal populations [26] (see S1 File for details). Here, unlike the EB smoothing, the rates are not smoothed but rather transformed into a new standardized variable. We computed the global and local Moran’s indices using the EB standardized variable.

For the global Moran’s *I*, we used the Monte Carlo simulations to generate the empirical frequencies to test the null hypothesis of no spatial autocorrelation. We generated 999 independent permutations of the EB standardized variable and computed *I* for each permutated vector to generate the empirical distribution. The *p*-value was estimated as the proportion of the number of times the indices from the permuted data exceeds the index from the actual data. For the LISA statistics, we used the Saddlepoint approximation method [27] to estimate the *p*-values due to the small number of neighbors of districts.

### Modeling the temporal patterns

To evaluate the temporal trends, we fitted time series exploratory models with autoregressive and moving average terms. The Poisson assumption of equal mean and variance is rarely met. Hence, conditional on the serial dependence in the response process, we expressed *y*_*t*_ as samples drawn from the negative-binomial distribution, *y*_*t*_|*ξ*_*t*_, *λ*_*t*_ = *NegBin*(*λ*_*t*_, *α*). For this, *α* is the over-dispersion parameter of which $\sigma_{y}^{2}=1/\alpha$ is the dispersion coefficient. We modeled the serial correlations *ξ*_*t*_ as linear combination of past predictive Pearson’s residuals *e*_*t*−*i*_ and latent means *λ*_*t*−*i*_; *log*(*λ*_*t*_) = *β*_*0*_ + *ξ*_*t*_, where $\xi_{t}=\sum_{p=1}^{P}\varphi_{p}(\xi_{t-p}+\varepsilon_{t-p})+\sum_{q=1}^{Q}\theta_{q}(\varepsilon_{t-q})$ is an autoregressive moving average recursion which induces serial correlation. Here *φ*_*p*_ and *θ*_*q*_ are the autoregressive and moving average parameters of orders *P* and *Q*, respectively. Following [21], the minimum element of the set *q* should be chosen in such a way that it is larger than the maximum element of *p* to avoid errors. For ease of interpretation we chose *p* = {1,2} and *q* = 3 to obtain the first and second-order autoregressive terms ***φ*** = {*φ*_1_, *φ*_2_} and three-month moving average term *θ*_3_. We fitted Poisson (**Model 1**) and Negative Binomial (**Model 2**) autoregressive moving average models. The models were fitted for the cumulative incidences for the entire country and each of the ten regions in our study area. We estimated the model parameters by maximum likelihood using the *glarma* package [21] of the R statistical software [28]. We compared the models fit using the Akaike Information Criterion (AIC) [29].

## Results and analyses

### Spatial distribution maps

**Fig 2:**
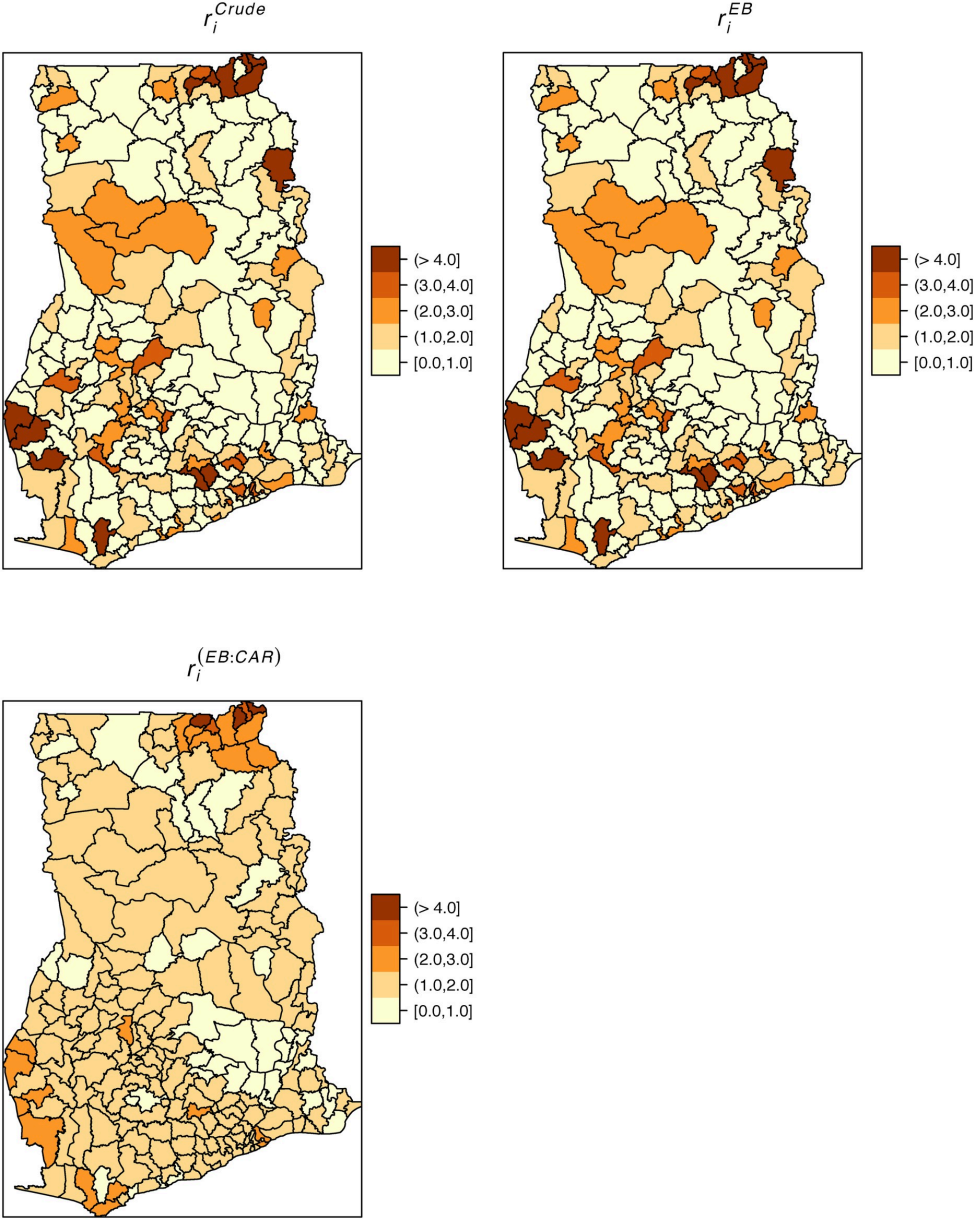
Crude, EB and CAR smoothed average rates. These maps show the crude, EB and CAR smoothed average typhoid rates per 100 people from 2011–2015. These maps were created using R statistical software.

We derived smoothed estimates of district-level typhoid rates for the years 2011 to 2015. Fig 2 shows the choropleth maps of the crude, EB smoothed, and the CAR smoothed rates per 100 persons from 2011 to 2015. The EB rates for the sparsely populated districts, especially those within the northern and central parts were shrunk towards their local averages, while those for highly populated areas hardly changed. After categorization, the crude and EB smoothed rates are barely distinguishable since smoothing did not cause changes in the class intervals. The CAR smoothed map, however, is easily distinguishable from the crude and the EB smoothed maps. The CAR map is smoother and shows large homogenous spatial patterns than the crude and EB maps, a result expected due to its construction.

**Fig 3:**
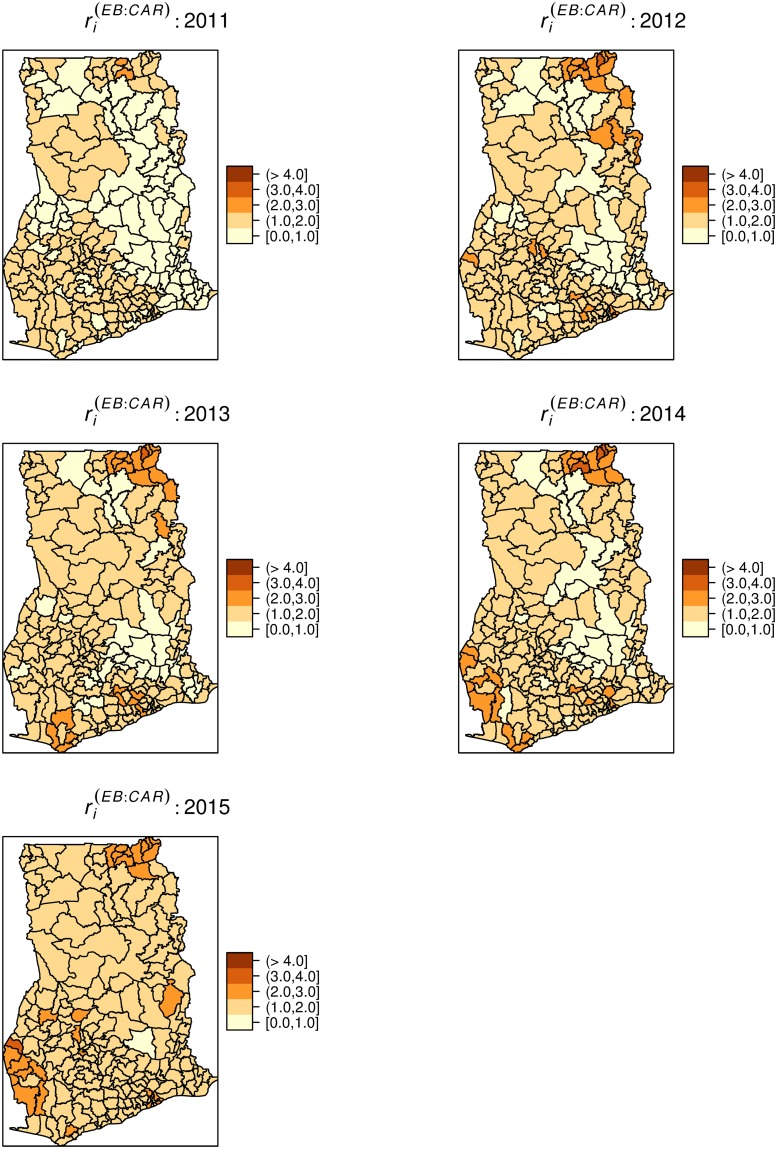
Yearly CAR smoothed typhoid rates. These maps show the yearly CAR smoothed typhoid rates per 100 people, 2011–2015. These maps were created using R statistical software.

Table 1 presents the summary statistics of the yearly crude, EB smoothed, and the CAR smoothed rates of typhoid. The yearly CAR smoothed rates are observed to have lower ranges compared with the crude and EB estimates. Fig 3 depicts the CAR smoothed maps of the spatial distribution of the yearly typhoid risks (see S1 File for maps of the crude rates). The spatial patterns are observably similar across the years under study as districts with high or low rates mostly persisted throughout the study period. The high rates prevailed within the north-eastern and west-central parts.

**Table 1:** This Table shows the summary statistics of the crude, EB smoothed, and the CAR smoothed rates of typhoid.

	Year				
	2011	2012	2013	2014	2015
**Summary**	***Crude rates***				
Minimum	0.012	0.010	0.010	0.018	0.013
1st Quartile	0.222	0.265	0.352	0.399	0.426
Median	0.676	0.708	0.847	0.929	1.084
Mean	1.095	1.425	1.460	1.454	1.684
3rd Quartile	1.562	1.927	1.942	1.807	2.220
Maximum	7.523	10.149	11.522	9.465	15.062
	***EB smoothed rate***				
Minimum	0.010	0.010	0.010	0.02	0.020
1st Quartile	0.220	0.267	0.357	0.4	0.430
Median	0.680	0.710	0.850	0.93	1.085
Mean	1.096	1.425	1.460	1.454	1.684
3rd Quartile	1.562	1.930	1.945	1.802	2.223
Maximum	7.520	10.15	11.52	9.46	15.06
	***CAR smoothed rates***				
Minimum	0.555	0.399	0.431	0.422	0.957
1st Quartile	0.934	1.117	1.152	1.152	1.458
Median	1.073	1.380	1.402	1.372	1.621
Mean	1.095	1.425	1.460	1.454	1.684
3rd Quartile	1.214	1.628	1.652	1.601	1.830
Maximum	2.141	3.067	3.323	3.300	3.065

### Spatial patterns

**Fig 4:**
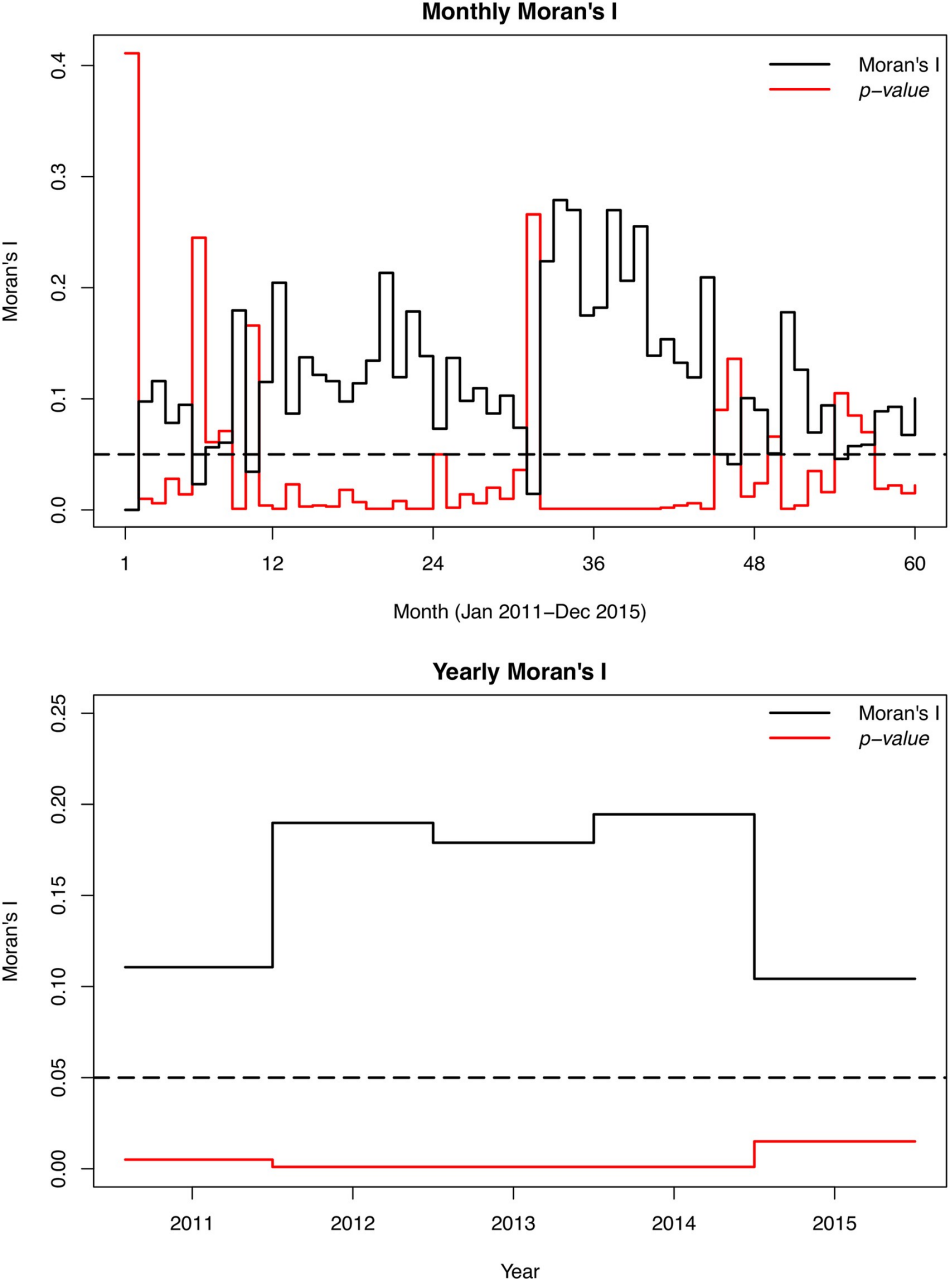
Monthly and yearly global Moran’s *I*. Step-graphs (Black steps) of global Moran’s *I* for monthly (above) and yearly (below) typhoid rates and the associated *p*-values (Red steps). Dashed line indicates significant level at *p* = 0.05. The monthly indices are from January 2011 (1) to December 2015 (60).

We estimated global Moran’s *I* for each *Month* = 1,…, 60, *Year* = 1,…, 5, and cumulative for the 5 years. Fig 4 shows the monthly (top) and yearly (down) Moran’s *I* and their associated *p*-values, respectively. The spatial distribution of typhoid risk was largely clustered (0 ≤ *I* ≤ 1). The monthly indices of clustering ranged from ≈ 0 − 0.28, while the yearly estimates ranged from 0.1 to 0.19. For the monthly spatial associations, 48 out of the 60 months showed significant spatial clustering at *p* < 0.05. The yearly spatial associations were all significant at *p* < 0.05. The overall index of spatial association was 0.19 (*p* < 0.001).

**Fig 5:**
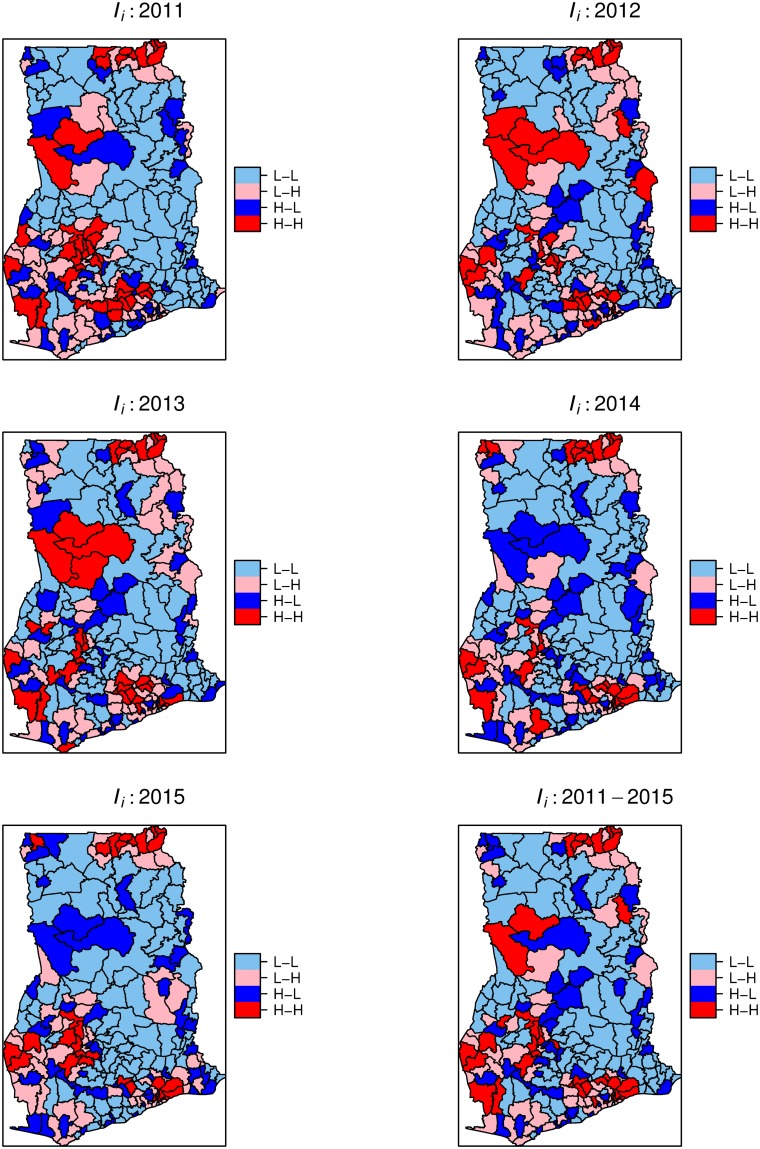
Local Moran’s *I* cluster maps. Local Moran’s *I* cluster maps showing high-high (H-H), low-low (L-L), low-high (L-H), and high-low (H-L) spatial associations. The maps were created using R statistical software.

**Fig 6:**
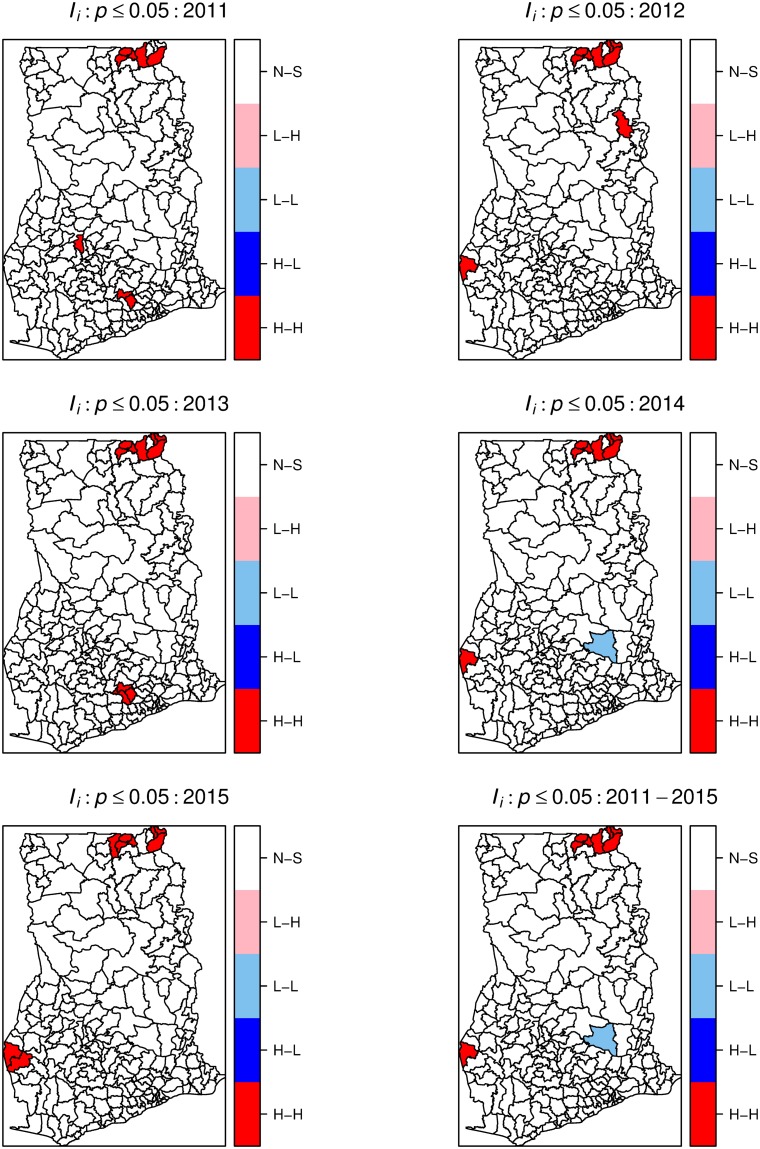
Local Moran’s *I* significant maps. Local Moran’s *I* significant maps showing (*p* < 0.05) high-high (H-H), low-low (L-L), low-high (L-H), and high-low (H-L) spatial associations. The maps were created using R statistical software.

We derived yearly and cumulative local Moran’s quadrant maps to indicate local areas with high-high (H-H), low-low (L-L), high-low (H-L), and low-high (L-H) clusters (Fig 5). The H-H and L-L categories indicate clustering of high rates (hot-spot) and low rates (cold-spots), respectively, equivalent to a positive spatial association. The H-L and L-H categories represent spatial outliers, local areas with a mixture of high and low rates in neighboring districts. Fig 6 shows the significant maps at *p* < 0.05. These maps indicate significant isolated but persistent hot-spots within the Upper-east region. We also observed isolated emerging and re-emerging hot-spots, mostly localized and occurring at the western parts. Comparing Figs 5 and 6, the cold-spots were persistent, but mostly not statistically significant. No significant spatial outliers were detected.

### Temporal patterns

**Fig 7:**
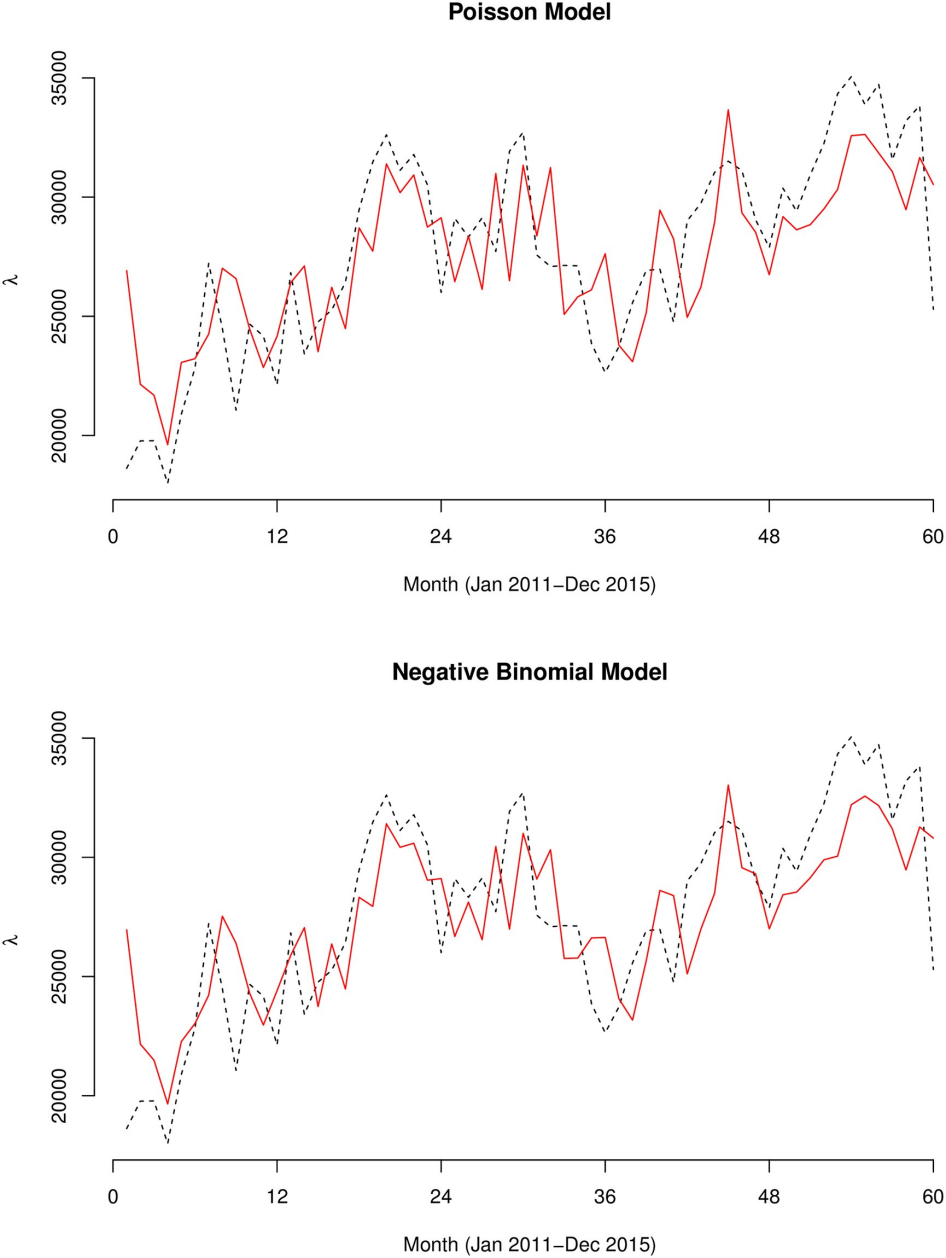
Graphs of the Poisson and Negative Binomial models. The observed (black dashed line) and predicted (red smooth line) counts of the Poisson (above) and Negative Binomial (below) generalized linear autoregressive moving average models for the whole of Ghana.

**Fig 8:**
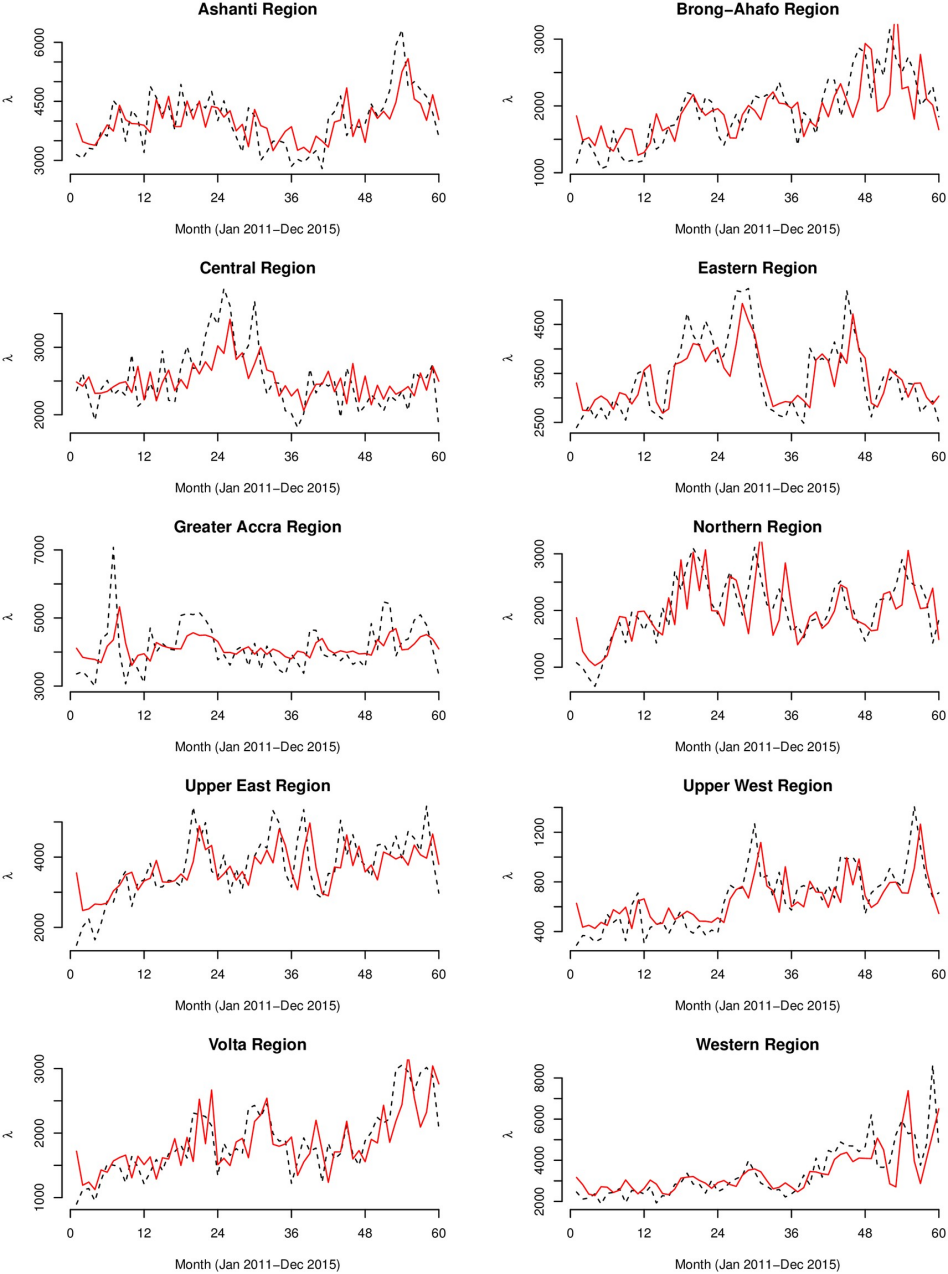
Regional graphs of the Poisson models. The observed (black dashed line) and predicted (red smooth line) counts of the Poisson generalized linear autoregressive moving average models for the 10 regions of Ghana.

**Fig 9:**
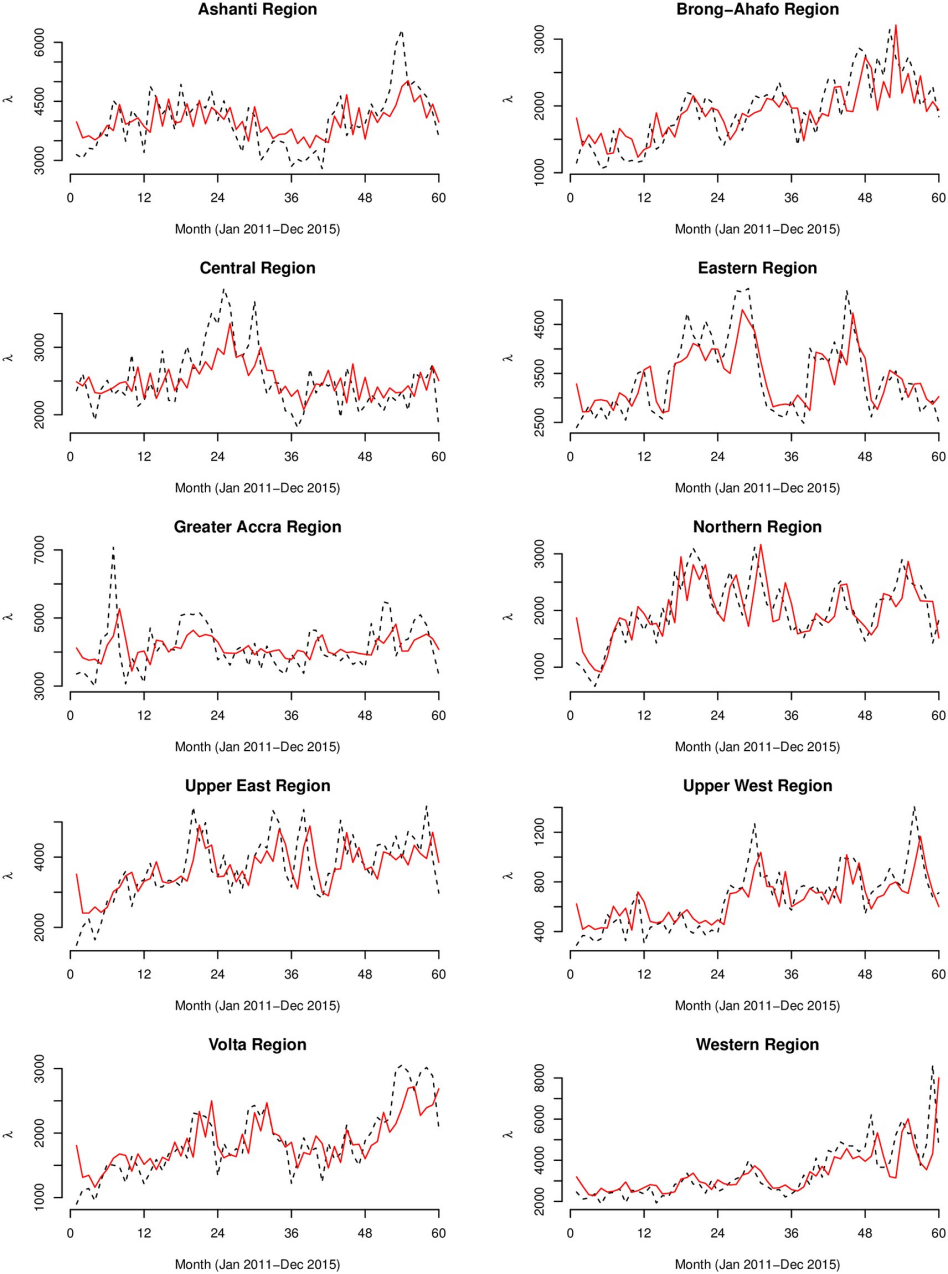
Regional graphs of the Negative Binomial models. The observed (black dashed line) and predicted (red smooth line) counts of the Negative Binomial (below) generalized linear autoregressive moving average models for the 10 regions of Ghana.

The fitted models of both the Poisson and Negative Binomial models are shown in Figs 7–9. Summary statistics of the models are also shown in Table 2. The autocorrelation functions of the Pearson’s residuals indicate that the models have dealt adequately with any serial correlation present (see S1 File). At *p* < 0.01 for all the parameters, the null hypothesis of no serial and moving average dependence cannot be accepted at 1% significant level as the estimated *z* − *scores* for all model parameters were greater than 2.7. In comparing the fit of the models, the Negative Binomial models had far lower AIC values than the Poisson models, hence, considered more satisfactory. For instance, the AIC for the country model of Model 1 drastically reduced from 17959.14 to 1129.79 for Model 2. Additional grounds for the choice of the negative Binomial models over the Poisson models is the degree of over-dispersion. The over-dispersion coefficients $\sigma_{y}^{2}=1/\alpha$ were significantly different from zero. For instance, for the country model of Model 2, *α* = 91.30, implying $\sigma_{y}^{2}=0.011$ which is 99% significantly different from zero (*z* − *score* = 4.5). Unlike the models of Model 1, the models of Model 2 captured significant multiplicative effects of first and second-order serial dependence. For the overall model, the multiplicative effects of the previous and two-time units back on the current incidences were $e^{\varphi_{1}}=7.25\%$ and $e^{\varphi_{2}}=4.64\%$, respectively. Additionally, we observed significant quarterly increments of $e^{\theta_{3}}=6.67\%$ by regressing on the person’s residuals three-time units back *ε*_*t*−3_. We observed remarkable first and second-order serial dependences of $e^{\varphi_{1}}=\{21.15\%,20.91\%\}$ and $e^{\varphi_{2}}=\{12.1\%,14.2\}$ for the Upper West and Northern regions, respectively. These same regions also featured amongst the highest three-time units back moving average dependencies $e^{\theta_{3}}=\{11.98\%,7.59\%\}$, respectively.

**Table 2:** This Table shows the estimates of parameters in the Poisson and Negative Binomial *glarma* models.

	Model 1: Poisson GLARMA					Model 2: Negative Binomial GLARMA					
	*β*_0_	$e^{\varphi_{1}}$	$e^{\varphi_{2}}$	$e^{\theta_{3}}$	*AIC*	*β*_0_	$e^{\varphi_{1}}$	$e^{\varphi_{2}}$	$e^{\theta_{3}}$	*α*	*AIC*
***Country models***	10.20	0.39	0.30	0.42	17959.14	10.18	7.25	4.64	6.75	91.30	1129.79
***Regional models***											
Ashanti	8.28	0.99	0.55	0.73	5231.02	8.28	6.75	4.10	8.35	52.86	935.06
Brong-Ahafo	7.53	1.34	0.65	0.92	5562.22	7.52	12.98	7.34	8.26	26.19	879.29
Central	7.82	0.98	0.25	0.77	3628.56	7.82	6.87	1.30	5.20	50.16	880.66
Eastern	8.10	1.17	0.99	0.65	4915.87	8.10	10.80	8.00	4.56	47.90	920.27
Greater Accra	8.32	0.57	0.32	0.07	6737.47	8.32	5.74	4.12	1.54	38.17	952.91
Northern	7.54	2.13	1.79	1.20	6161.76	7.54	20.93	14.20	7.59	23.25	893.11
Upper East	8.17	1.05	0.70	0.46	9633.26	8.16	14.49	8.20	4.38	23.76	970.92
Upper West	6.44	2.75	1.75	1.54	3250.12	6.43	21.15	12.10	11.98	15.33	787.05
Volta	7.45	1.87	1.43	1.69	5030.55	7.44	11.85	10.22	14.48	27.72	880.16
Western	8.06	0.92	1.38	0.83	11756.41	8.03	13.85	14.46	3.64	22.95	964.97

## Discussion

We observed several notable findings. From the CAR smoothed map, there are substantial spatial patterns, which is suggestive of spatially varied causal factors of similar patterns wealthy of further investigation. There were also observable similar spatial patterns across the years under study probably because the causal risk factors have been less dynamic over the years. These developed maps could be resourceful for guiding and prioritizing interventions such as vaccination. The observance of similar spatial patterns is also indicative of consistent sources of infection or causal factors. These maps could serve as starting points for further etiological investigation, and monitoring, planning, and evaluation of interventions.

Since typhoid transmission is dependent upon contact with faecally contaminated water or food [1,2] which depends on environmental sanitation conditions which are themselves spatially continuous in nature, we expected a higher tendency of clustering. Primarily, the global spatial autocorrelation analysis found strong evidence of typhoid occurrences being influenced by incidences from neighboring districts, varying yearly and monthly. This knowledge can guide public health professionals in their search for possible interventions. The nonrandom spatial distribution reaffirms the idea of the involvement of environmental casual factors and instigates an etiological clue that the possible causal factors are spatially structured. These patterns are characteristic of typhoid as comparable patterns have been observed by similar studies in the Zhejiang Province of China [8] and the Dhaka metropolitan area of Bangladesh [30]. In a population-based study in an urban area of Nairobi, Kenya, Akullian et al [31] associated the geographical distribution of typhoid to lower elevation. Further steps in search of etiological clues through the building of association models will be worthwhile.

The local Moran’s *I* maps indicated fewer hot-spots, almost repeating throughout the study period, suggesting a focalized nature of typhoid transmission in Ghana. The developments of these maps are particularly important as they can be relevant for evaluating etiological characteristics of the disease. Of particular interest in such maps is the assessment of the correlation between etiological factors and the hot-spots which we seek to investigate in a follow-up study. The persistent hop-spots within the Upper East region and the emerging and re-emerging ones within the Western region suggest that vaccination policy, if any, should prioritize these areas. Additional intervention programs requiring prioritization in these areas should include public health education, provision of safe drinking water and sanitation. The general consistency of the hot-spots in our study contrasts with historical clustering dynamics of typhoid in Washington DC-USA as there was a general lack of consistent re-emerging clusters between outbreak years [32]. This contrast may suggest different modes of transmission and risk factors between developing and developed countries. The consistent and re-emergence of hot-spot in these areas may be attributed to the unique characteristics of the low level of education, high poverty, poor housing, poor drinking water, and poor sanitation within these regions [33]. These findings also suggest poverty alleviation programs and provision of amenities such safe drinking water and sanitation should be directed towards these areas. Currently, to the best of our knowledge, there has been no risk factor identification studies of typhoid in Ghana. That notwithstanding, our suspicion of sociodemographic risk factors such as low level of education, high poverty, poor housing, poor drinking water, and poor sanitation has been confirmed elsewhere [34].

The *glarma* models suggest distinct temporal patterns with significant first and second order serial dependencies. These results underline the relevance of generalized autoregressive moving average models in explaining the temporal dynamics of infectious diseases. This knowledge is important as it prompts for further research to evaluate important climatic variables that modulate typhoid incidences. We observed significant first and second-order serial dependencies which are indicative of systematic linear trend increments. This is possibly influenced by climate-related variables that may modulate the incidences of typhoid. An additional explanation may include true increases in incidences due to deteriorating water and sanitation systems resulting from high population growths, or mere improvements in reporting and surveillance systems. Elsewhere, higher incidences of typhoid have been associated with high population densities [30], hence temporal increases in population density can effectively increase incidences. The serial correlations varied across the different regions, indicating the possible influence of regional level climatic factors or variations of improvements in disease surveillance. Climatic variables, such as rainfall, vapor pressure, and temperature, have been indicated as having an important effect on the transmission and distribution of typhoid infections in Vietnam [35,36] and China [35,36]. Ghana has a complex spatial variability of rainfall patterns. For instance, the northern parts experience a single wet season occurring between May and November, while the southern parts have two wet seasons: the major season from March to July, and a minor season from September to November [37]. In Bangladesh, Corner et al [34] inferred spatial variation of increased temperature to increase the risk of typhoid using land surface temperature (LST). In fact, typhoid fever has been observed to be sensitive to changes in either one or more of climate-related variables like temperature, precipitation and absolute humidity [38,39].

In Ghana, multi-locational analysis of communicable disease epidemiology is rare. Our use of district-level morbidity data presents an advantage to access spatial variation in the disease risk. While neighborhood health planning mainly dwells on administrative districts, this study is relevant to complement neighborhood health planning in Ghana. For typhoid, this study is the first to demonstrate the country-wide spatial and temporal epidemiology. We have demonstrated that district-level variation of typhoid incidences is spatially clustered with varying monthly and yearly magnitudes. An additional strength of this study is the separate *glarma* models for each of the 10 regions, which allowed us to compare the serial and seasonal dependencies among regions.

The interpretation of our results should be in light of certain limitations. First, our data were aggregated at districts levels, hence, we make inferences at the group level rather than the individual level to avoid issues of ecological fallacy [19]. Lack of diagnostics laboratories in certain reporting facilities may cause variation in reporting patterns. In fact, overlap symptoms between typhoid and malaria could possibly lead to underreporting of typhoid cases because many typhoid incidences are likely to be misdiagnosed as malaria [40]. We have not considered the spatial correlation between each of the times series models; we endeavor to follow up this study with a multivariate times series model which could account for such correlations. The CAR mapping implicitly assumes that spatial variation is only due to spatial correlation. Our further study seeks to develop fully Bayesian models that could account for both covariates variation and spatial correlation.

## Conclusion

We conclude that both the global and local Moran’s spatial autocorrelation were able to detect the clustering patterns and district-level contributions to the patterns. We have demonstrated that district-level variation of typhoid incidences is spatially clustered with varying monthly and yearly magnitudes, an indication that incidences are influenced by incidences from adjacent districts. Higher than expected risks of typhoid occur among several districts within the Upper West and Western regions, with occasional emerging and re-emerging ones. The negative binomial generalized linear autoregressive moving average models were found to afford adequate fit to the data. Serial correlations indicate temporal dependencies of typhoid risk with incidences in the previous months, showing varying magnitudes across the different regions. The study clearly shows that spatial and temporal statistical analysis of reported morbidity data can be useful to gain insight into the variation of typhoid fever in space and time.

## Supporting information

S1 File Supporting information. This file contains a detailed mathematical description of the statistical methods, additional Figures and sample data.
(DOCX)
