# Supplementary material for: Spatial and temporal heterogeneities of district-level typhoid morbidities in Ghana: A requisite insight for informed public health response
Source: PLoS One. 2018 Nov 29;13(11):e0208006. doi: 10.1371/journal.pone.0208006 (PMC6264858; doi:10.1371/journal.pone.0208006)
Supplement: S1 File — This file contains a detailed mathematical description of the statistical methods, additional Figures and sample data. (DOCX) [file pone.0208006.s001.docx]

**Appendix**

**Empirical Bayesian smoothing**

The smoothed estimates of the relative risks are expressed as $r_{it}^{EB}=\varpi_{it}r_{it}+\left( 1-\varpi_{it} \right)\bar{r}_{it}$, where $\varpi_{it}={\sigma_{it}^{2}}/\left\{ \sigma_{it}^{2}+\left( {\bar{r}_{it}}/{n_{it}} \right) \right\}$ are the respective weights. Here $\bar{r}_{it}$ and $\sigma_{it}^{2}$ are the spatially varying estimates for the prior mean and variance, respectively. We used the method of moments to estimate $\bar{r}_{it}={\sum_{j} w_{ij}y_{it}}/{\sum_{j} w_{ij}n_{it}}$ and $\sigma_{it}^{2}=\left\{ {\sum_{j} w_{ij}n_{it}\left( r_{it}-\bar{r}_{it} \right)^{2}}/{\sum_{j} w_{ij}n_{it}} \right\}-\left\{ {\bar{r}_{it}}/{\sum_{j} w_{ij}n_{it}} \right\}$ [22]. We defined the spatial neighborhood structure $w_{ij}$ as a binary connectivity weight matrix; $w_{ij}=1$ if $\left( i \right)\bigcap\left( j \right)\neq null$, and 0 otherwise, where $\left( i \right)$and $\left( j \right)$ are the set of boundary points of district $i$ and $j$, respectively. The $m$ by $m$ weight matrix $w_{ij}$ was row-standardized and satisfied the following conditions (1) symmetry, i.e., $w_{ij}=w_{ji}$, (2) zero diagonal elements by convention to prevent an observation from predicting itself, i.e. $w_{ii}=0$, and (3) normalization, i.e., $\sum_{j} w_{ij}=1$.

**Empirical Bayesian Standardization**

We constructed an empirical Bayes standardized variate $z_{i}^{EB}=\left( r_{i}-\tilde{r} \right)/{\sqrt{v_{i}}}$ based upon the unconditional marginal expectation of the risk $\tilde{r}$ and district-specific variance $v_{i}=s^{2}+\tilde{r}/{n_{i}}$. The method of moments estimates for $s^{2}=\sigma^{2}-\tilde{r}/\left( {\sum n_{i}}/m \right)$, where $\sigma^{2}={\sum n_{i}\left( r_{i}-\tilde{r} \right)^{2}}/{\sum n_{i}}$. We computed the global Moran’s $I=\sum_{i} \sum_{j} w_{ij}z_{i}^{EB}z_{j}^{EB}$ and local Moran’s $I_{i}=z_{i}^{EB}\sum_{j} w_{ij}z_{j}^{EB}$ using the standardized variable $z_{i}^{EB}$. For the local indices, $I_{i}$, the summation over *j* implies that only the set of neighbors $J_{i}$ of *i*, $j\in J_{i}$, is included.


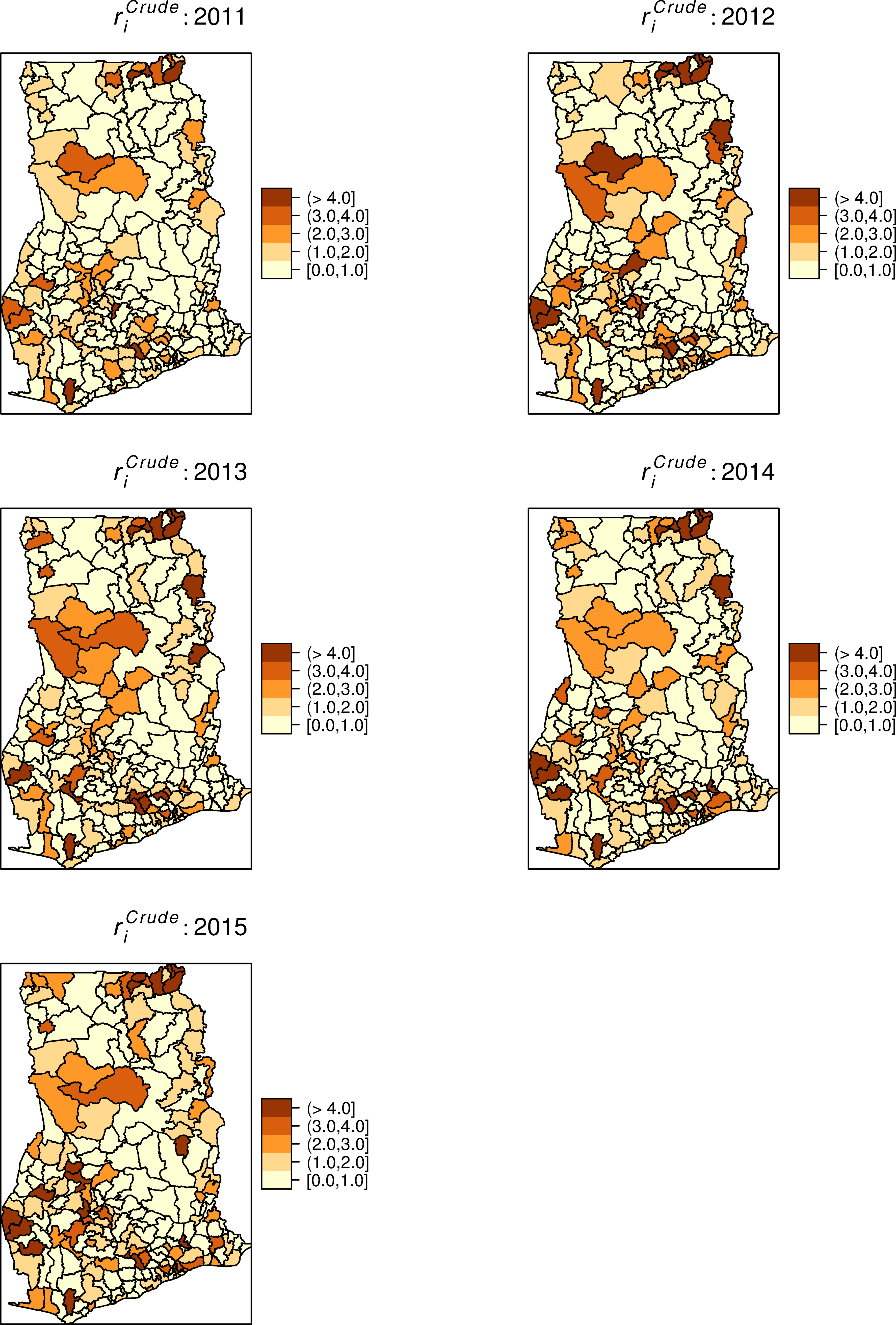


**Fig A1**. Yearly typhoid rates per 100 people, 2011-2015. These maps were created using R statistical software

**Fig A2**. Autocorrelation functions of the person’s residuals of the Negative Binomial generalized autoregressive moving average models for the whole of Ghana.

**Fig A3**. Autocorrelation functions of the person’s residuals of the Negative Binomial generalized autoregressive moving average models for the 10 regions of Ghana.

**Fig A4**. Autocorrelation functions of the person’s residuals of the Negative Binomial generalized autoregressive moving average models for the 10 regions of Ghana.

Table A1: Sample Data

| **Region** | **District** | **ID** | **Pop** | **mon_1** | **mon_2** | **mon_3** | **mon_4** | **mon_5** | **mon_6** | **mon_7** | **mon_8** | **mon_9** | **mon_10** | **mon_11** | **mon_12** |
| --- | --- | --- | --- | --- | --- | --- | --- | --- | --- | --- | --- | --- | --- | --- | --- |
| Central | Abura-Asebu-Kwamankese | 1 | 117185 | 16 | 18 | 21 | 41 | 29 | 57 | 31 | 57 | 28 | 63 | 49 | 23 |
| Greater Accra | Accra Metro | 2 | 1665086 | 1059 | 719 | 607 | 757 | 1343 | 1045 | 1271 | 879 | 846 | 977 | 982 | 783 |
| Greater Accra | Ada East | 3 | 71671 | 23 | 29 | 29 | 35 | 78 | 54 | 42 | 19 | 22 | 12 | 11 | 14 |
| Greater Accra | Ada West | 4 | 59124 | 37 | 26 | 13 | 17 | 27 | 27 | 9 | 44 | 3 | 16 | 13 | 8 |
| Volta | Adaklu | 5 | 36391 | 0 | 0 | 0 | 0 | 0 | 0 | 0 | 0 | 0 | 0 | 0 | 0 |
| Ashanti | Adansi North | 6 | 107091 | 8 | 14 | 14 | 1 | 15 | 0 | 3 | 6 | 12 | 11 | 10 | 13 |
| Ashanti | Adansi South | 7 | 115378 | 34 | 60 | 79 | 41 | 50 | 8 | 19 | 22 | 33 | 25 | 32 | 17 |
| Greater Accra | Adentan | 8 | 78215 | 195 | 202 | 257 | 213 | 225 | 289 | 297 | 247 | 154 | 164 | 107 | 81 |
| Volta | Afadjato South | 9 | 95030 | 2 | 16 | 4 | 1 | 5 | 20 | 8 | 27 | 19 | 36 | 15 | 11 |
| Ashanti | Afigya-Kwabre | 10 | 136140 | 122 | 122 | 50 | 235 | 219 | 78 | 233 | 257 | 175 | 172 | 268 | 194 |
| Ashanti | Sekyere South | 11 | 94009 | 132 | 174 | 116 | 156 | 152 | 175 | 204 | 125 | 89 | 86 | 158 | 83 |
| Central | Agona East | 12 | 85920 | 22 | 13 | 33 | 39 | 54 | 65 | 76 | 61 | 18 | 57 | 46 | 17 |
| Central | Agona West | 13 | 115358 | 98 | 79 | 64 | 31 | 39 | 10 | 132 | 30 | 21 | 39 | 18 | 40 |
| Volta | Agortime-Ziope | 14 | 28013 | 1 | 0 | 0 | 0 | 2 | 3 | 3 | 5 | 2 | 2 | 0 | 0 |
| Ashanti | Ahafo-Ano North | 15 | 94285 | 26 | 30 | 28 | 24 | 18 | 24 | 0 | 5 | 32 | 24 | 20 | 19 |
| Ashanti | Ahafo-Ano South | 16 | 121659 | 113 | 189 | 91 | 112 | 95 | 122 | 101 | 98 | 88 | 133 | 112 | 115 |
| Western | Ahanta West | 17 | 106215 | 121 | 93 | 101 | 70 | 104 | 116 | 132 | 70 | 62 | 62 | 53 | 146 |
| Central | Ajumako-Enyan-Essiam | 18 | 138046 | 43 | 57 | 74 | 53 | 65 | 81 | 124 | 42 | 66 | 121 | 111 | 122 |
| Volta | Akatsi North | 19 | 29777 | 0 | 0 | 0 | 0 | 0 | 0 | 0 | 0 | 0 | 0 | 0 | 0 |
| Volta | Akatsi South | 20 | 98684 | 6 | 126 | 67 | 59 | 50 | 6 | 30 | 13 | 62 | 92 | 83 | 76 |
| Eastern | Akwapim North | 21 | 136483 | 82 | 50 | 149 | 53 | 137 | 71 | 128 | 149 | 232 | 93 | 97 | 47 |
| Eastern | Akwapim South | 22 | 37501 | 0 | 10 | 9 | 12 | 0 | 4 | 52 | 35 | 13 | 13 | 33 | 14 |
| Eastern | Akyemansa | 23 | 97374 | 63 | 45 | 58 | 37 | 84 | 26 | 34 | 23 | 13 | 54 | 447 | 57 |
| Ashanti | Amansie Central | 24 | 90741 | 3 | 5 | 0 | 5 | 6 | 6 | 9 | 17 | 5 | 13 | 19 | 30 |
| Ashanti | Amansie West | 25 | 134331 | 36 | 78 | 66 | 152 | 143 | 130 | 127 | 195 | 105 | 227 | 222 | 226 |
| Western | Aowin | 26 | 117886 | 141 | 182 | 245 | 141 | 153 | 121 | 177 | 179 | 171 | 223 | 134 | 78 |
| Ashanti | Asante-Akim Central | 27 | 71508 | 681 | 346 | 342 | 260 | 325 | 446 | 571 | 300 | 260 | 287 | 120 | 75 |
| Ashanti | Asante-Akim North | 28 | 69186 | 9 | 3 | 7 | 4 | 15 | 5 | 19 | 9 | 10 | 20 | 9 | 17 |
| Ashanti | Asante-Akim South | 29 | 117245 | 27 | 39 | 48 | 41 | 31 | 46 | 41 | 26 | 41 | 0 | 5 | 1 |
| Greater Accra | Ashaiman | 30 | 190972 | 580 | 542 | 917 | 450 | 735 | 1036 | 2769 | 633 | 571 | 1003 | 739 | 454 |
| Central | Asikuma-Odoben-Brakwa | 31 | 112706 | 130 | 115 | 168 | 192 | 165 | 212 | 137 | 106 | 114 | 185 | 148 | 166 |
| Ashanti | Asokore-Mampong | 32 | 304815 | 0 | 10 | 20 | 0 | 0 | 0 | 0 | 0 | 0 | 0 | 20 | 0 |
| Central | Assin North | 33 | 161341 | 118 | 141 | 205 | 159 | 246 | 316 | 331 | 234 | 294 | 311 | 230 | 299 |
| Central | Assin South | 34 | 104244 | 342 | 284 | 303 | 356 | 434 | 256 | 3 | 0 | 0 | 306 | 120 | 77 |
| Brong Ahafo | Asunafo North | 35 | 124685 | 5 | 170 | 229 | 354 | 108 | 178 | 82 | 6 | 52 | 8 | 63 | 1 |
| Brong Ahafo | Asunafo South | 36 | 95580 | 8 | 3 | 4 | 5 | 14 | 26 | 11 | 28 | 8 | 39 | 3 | 31 |
| Eastern | Asuogyaman | 37 | 98046 | 120 | 13 | 14 | 13 | 23 | 6 | 21 | 23 | 39 | 35 | 30 | 17 |
| Brong Ahafo | Asutifi North | 38 | 52259 | 58 | 73 | 144 | 104 | 64 | 84 | 329 | 370 | 159 | 238 | 222 | 187 |
| Brong Ahafo | Asutifi South | 39 | 53584 | 120 | 102 | 144 | 1 | 2 | 18 | 0 | 119 | 1 | 1 | 0 | 1 |
| Brong Ahafo | Atebubu-Amanten | 40 | 105938 | 44 | 51 | 27 | 83 | 43 | 75 | 118 | 90 | 325 | 159 | 46 | 59 |
| Eastern | Atiwa | 41 | 110622 | 63 | 222 | 93 | 117 | 322 | 81 | 223 | 86 | 114 | 114 | 12 | 793 |
| Ashanti | Atwima-Kwanwoma | 42 | 90634 | 32 | 85 | 65 | 81 | 61 | 149 | 187 | 129 | 141 | 278 | 160 | 53 |
| Ashanti | Atwima-Mponua | 43 | 119180 | 0 | 0 | 0 | 0 | 0 | 0 | 0 | 0 | 0 | 2 | 3 | 0 |
| Ashanti | Atwima-Nwabiagya | 44 | 149025 | 245 | 171 | 238 | 191 | 333 | 277 | 454 | 417 | 397 | 367 | 409 | 403 |
| Central | Awutu Senya | 45 | 86884 | 102 | 70 | 69 | 54 | 56 | 65 | 153 | 163 | 145 | 210 | 280 | 286 |
| Central | Awutu Senya East | 46 | 108422 | 82 | 91 | 74 | 52 | 96 | 76 | 128 | 63 | 44 | 67 | 48 | 56 |
| Eastern | Ayensuano | 47 | 77193 | 0 | 0 | 0 | 2 | 0 | 3 | 0 | 0 | 3 | 0 | 0 | 0 |
| Brong Ahafo | Banda | 48 | 20282 | 1 | 0 | 7 | 0 | 0 | 0 | 0 | 3 | 1 | 3 | 0 | 0 |
| Upper East | Bawku | 49 | 98538 | 217 | 126 | 92 | 207 | 267 | 181 | 218 | 581 | 404 | 153 | 140 | 381 |
| Upper East | Bawku West | 50 | 94034 | 228 | 217 | 306 | 228 | 243 | 345 | 349 | 351 | 177 | 333 | 230 | 213 |
| Ashanti | Bekwai | 51 | 118024 | 130 | 168 | 226 | 74 | 122 | 119 | 116 | 115 | 88 | 126 | 94 | 87 |
| Brong Ahafo | Berekum | 52 | 129628 | 95 | 65 | 90 | 119 | 121 | 9 | 127 | 107 | 50 | 69 | 63 | 84 |
| Western | Bia East | 53 | 27393 | 16 | 5 | 5 | 23 | 8 | 15 | 7 | 13 | 7 | 11 | 2 | 10 |
| Western | Bia West | 54 | 88939 | 336 | 294 | 391 | 336 | 60 | 228 | 230 | 350 | 182 | 155 | 258 | 435 |
| Volta | Biakoye | 55 | 65901 | 43 | 10 | 7 | 32 | 57 | 34 | 1 | 40 | 65 | 25 | 133 | 30 |
| Upper East | Binduri | 56 | 61576 | 0 | 0 | 0 | 1 | 0 | 0 | 0 | 0 | 1 | 0 | 0 | 0 |
| Eastern | Birim Central | 57 | 144869 | 568 | 525 | 702 | 654 | 726 | 553 | 355 | 151 | 429 | 994 | 839 | 730 |
| Eastern | Birim North | 58 | 78907 | 8 | 18 | 7 | 10 | 13 | 14 | 25 | 32 | 26 | 31 | 23 | 23 |
| Eastern | Birim South | 59 | 119767 | 140 | 74 | 171 | 128 | 105 | 151 | 133 | 84 | 135 | 100 | 127 | 142 |
| Western | Bodi | 60 | 53314 | 7 | 9 | 44 | 5 | 0 | 0 | 5 | 12 | 2 | 4 | 4 | 2 |
| Northern | Bole | 61 | 61593 | 32 | 10 | 74 | 8 | 54 | 35 | 40 | 87 | 18 | 65 | 153 | 246 |
| Upper East | Bolgatanga | 62 | 131550 | 215 | 431 | 663 | 338 | 425 | 697 | 695 | 907 | 1023 | 882 | 1172 | 1107 |
| Upper East | Bongo | 63 | 84545 | 154 | 228 | 90 | 70 | 134 | 90 | 105 | 142 | 230 | 128 | 250 | 413 |
| Ashanti | Bosome-Freho | 64 | 60397 | 0 | 0 | 0 | 0 | 0 | 0 | 0 | 4 | 0 | 2 | 0 | 0 |
| Ashanti | Bosomtwi | 65 | 93910 | 24 | 19 | 51 | 56 | 4 | 20 | 4 | 9 | 16 | 0 | 11 | 31 |
| Upper East | Builsa North | 66 | 56477 | 105 | 55 | 108 | 86 | 93 | 158 | 148 | 233 | 237 | 261 | 223 | 174 |
| Upper East | Builsa South | 67 | 36514 | 0 | 0 | 25 | 10 | 21 | 15 | 71 | 130 | 62 | 90 | 79 | 34 |
| Northern | Bunkpurugu-Yunyoo | 68 | 122591 | 0 | 46 | 0 | 0 | 0 | 0 | 7 | 4 | 0 | 0 | 0 | 0 |
| Central | Cape Coast | 69 | 169894 | 579 | 841 | 390 | 299 | 326 | 436 | 346 | 460 | 706 | 768 | 450 | 279 |
| Volta | Central Tongu | 70 | 59411 | 14 | 6 | 6 | 0 | 10 | 4 | 5 | 2 | 3 | 1 | 0 | 19 |
| Northern | Chereponi | 71 | 53394 | 1 | 1 | 1 | 2 | 1 | 15 | 17 | 13 | 21 | 13 | 17 | 17 |
| Upper West | Daffiama-Bussie-Issa | 72 | 32827 | 0 | 0 | 1 | 0 | 0 | 0 | 0 | 0 | 5 | 0 | 9 | 0 |
| Eastern | Denkyembour | 73 | 78841 | 142 | 271 | 300 | 79 | 111 | 48 | 115 | 122 | 75 | 54 | 118 | 129 |
| Brong Ahafo | Dormaa East | 74 | 50871 | 0 | 0 | 0 | 0 | 0 | 0 | 0 | 0 | 0 | 0 | 0 | 0 |
| Brong Ahafo | Dormaa Municipal | 75 | 112111 | 109 | 86 | 94 | 94 | 72 | 107 | 150 | 64 | 70 | 108 | 105 | 170 |
| Brong Ahafo | Dormaa West | 76 | 47678 | 0 | 0 | 0 | 0 | 0 | 0 | 0 | 0 | 0 | 0 | 0 | 0 |
| Eastern | East Akim | 77 | 167896 | 152 | 146 | 43 | 151 | 90 | 184 | 216 | 192 | 128 | 219 | 256 | 137 |
| Northern | East Gonja | 78 | 135450 | 139 | 133 | 29 | 14 | 18 | 27 | 23 | 45 | 12 | 27 | 58 | 24 |
| Central | Efutu | 79 | 68597 | 97 | 33 | 61 | 102 | 104 | 64 | 28 | 115 | 16 | 50 | 36 | 70 |
| Ashanti | Ejisu-Juaben | 80 | 143762 | 190 | 183 | 213 | 192 | 189 | 164 | 285 | 308 | 203 | 226 | 212 | 219 |
| Ashanti | Ejura-Sekyedumase | 81 | 85446 | 195 | 142 | 161 | 144 | 169 | 173 | 172 | 248 | 266 | 261 | 275 | 215 |
| Central | Ekumfi | 82 | 52231 | 0 | 0 | 1 | 0 | 3 | 0 | 0 | 1 | 5 | 1 | 0 | 0 |
| Western | Ellembelle | 83 | 87501 | 208 | 75 | 98 | 105 | 162 | 152 | 207 | 229 | 197 | 254 | 231 | 203 |
| Eastern | Fanteakwa | 84 | 108614 | 4 | 11 | 30 | 40 | 0 | 18 | 32 | 48 | 32 | 32 | 33 | 32 |
| Greater Accra | Ga Central | 85 | 117220 | 73 | 201 | 228 | 120 | 169 | 157 | 21 | 170 | 124 | 29 | 167 | 76 |
| Greater Accra | Ga East | 86 | 147742 | 74 | 159 | 73 | 78 | 91 | 80 | 61 | 56 | 67 | 28 | 196 | 135 |
| Greater Accra | Ga South | 87 | 411377 | 188 | 189 | 193 | 256 | 317 | 315 | 539 | 393 | 161 | 121 | 348 | 78 |
| Greater Accra | Ga West | 88 | 219788 | 504 | 562 | 348 | 493 | 532 | 555 | 845 | 677 | 506 | 532 | 268 | 715 |
| Upper East | Garu-Tempane | 89 | 130003 | 306 | 525 | 514 | 324 | 430 | 602 | 338 | 590 | 738 | 271 | 752 | 566 |
| Central | Gomoa East | 90 | 207071 | 178 | 173 | 199 | 160 | 125 | 229 | 257 | 232 | 329 | 219 | 194 | 168 |
| Central | Gomoa West | 91 | 135189 | 40 | 49 | 81 | 52 | 53 | 62 | 79 | 81 | 67 | 52 | 47 | 46 |
| Northern | Central Gonja | 92 | 87877 | 24 | 33 | 67 | 47 | 73 | 340 | 406 | 422 | 257 | 318 | 266 | 154 |
| Northern | Gushiegu | 93 | 111259 | 6 | 5 | 2 | 9 | 16 | 9 | 5 | 12 | 12 | 10 | 7 | 1 |
| Volta | Ho | 94 | 177281 | 153 | 188 | 310 | 214 | 336 | 384 | 505 | 380 | 272 | 333 | 437 | 390 |
| Volta | Ho West | 95 | 94600 | 1 | 0 | 0 | 0 | 0 | 0 | 1 | 0 | 0 | 0 | 0 | 0 |
| Volta | Hohoe | 96 | 167016 | 25 | 70 | 40 | 1 | 1 | 23 | 40 | 18 | 11 | 0 | 12 | 5 |
| Brong Ahafo | Jaman North | 97 | 83059 | 31 | 52 | 19 | 19 | 35 | 16 | 52 | 35 | 11 | 12 | 16 | 21 |
| Brong Ahafo | Jaman South | 98 | 92649 | 218 | 152 | 83 | 60 | 71 | 130 | 129 | 59 | 30 | 40 | 82 | 38 |
| Volta | Jasikan | 99 | 59181 | 12 | 33 | 35 | 76 | 38 | 65 | 39 | 54 | 50 | 36 | 62 | 9 |
| Upper West | Jirapa | 100 | 88402 | 75 | 94 | 137 | 139 | 121 | 243 | 192 | 149 | 124 | 132 | 218 | 63 |
| Western | Jomoro | 101 | 150107 | 97 | 105 | 66 | 109 | 111 | 123 | 111 | 122 | 119 | 146 | 102 | 82 |
| Western | Juaboso | 102 | 58435 | 322 | 195 | 47 | 255 | 152 | 75 | 137 | 164 | 106 | 143 | 170 | 231 |
| Volta | Kadjebi | 103 | 59303 | 0 | 0 | 0 | 0 | 0 | 0 | 0 | 49 | 0 | 35 | 46 | 33 |
| Northern | Karaga | 104 | 77706 | 1 | 5 | 7 | 1 | 32 | 24 | 14 | 11 | 16 | 15 | 12 | 12 |
| Upper East | Kasena-Nankana | 105 | 70667 | 82 | 61 | 55 | 91 | 72 | 30 | 25 | 44 | 30 | 43 | 34 | 22 |
| Upper East | Kasena-Nankana West | 106 | 109944 | 47 | 74 | 43 | 67 | 75 | 377 | 75 | 110 | 115 | 120 | 84 | 47 |
| Volta | Keta | 107 | 147618 | 273 | 288 | 258 | 83 | 211 | 258 | 218 | 208 | 156 | 530 | 123 | 303 |
| Volta | Ketu North | 108 | 99913 | 14 | 44 | 69 | 26 | 34 | 36 | 42 | 29 | 35 | 20 | 54 | 41 |
| Volta | Ketu South | 109 | 160756 | 0 | 0 | 2 | 15 | 6 | 1 | 13 | 20 | 25 | 8 | 3 | 0 |
| Brong Ahafo | Kintampo North | 110 | 95480 | 36 | 88 | 66 | 28 | 41 | 20 | 26 | 43 | 38 | 40 | 49 | 26 |
| Brong Ahafo | Kintampo South | 111 | 81000 | 2 | 2 | 1 | 0 | 10 | 2 | 2 | 1 | 2 | 2 | 3 | 0 |
| Ashanti | Kumasi | 112 | 1730249 | 318 | 529 | 627 | 737 | 894 | 895 | 900 | 1037 | 906 | 1256 | 1077 | 818 |
| Central | Komenda-Edna-Eguafo-Abirem | 113 | 144705 | 58 | 65 | 114 | 56 | 78 | 97 | 67 | 245 | 66 | 62 | 54 | 48 |
| Northern | Kpandai | 114 | 108816 | 23 | 37 | 27 | 20 | 68 | 26 | 33 | 66 | 58 | 49 | 35 | 17 |
| Volta | Kpando | 115 | 53736 | 53 | 44 | 93 | 139 | 90 | 55 | 69 | 83 | 108 | 99 | 90 | 80 |
| Greater Accra | Kpone-Katamanso | 116 | 109864 | 15 | 5 | 10 | 11 | 16 | 10 | 1 | 0 | 7 | 0 | 5 | 0 |
| Volta | Krachi East | 117 | 116804 | 48 | 42 | 20 | 69 | 62 | 124 | 169 | 123 | 77 | 51 | 86 | 33 |
| Volta | Krachi Nchumuru | 118 | 72688 | 7 | 28 | 23 | 23 | 26 | 45 | 20 | 21 | 22 | 14 | 23 | 14 |
| Volta | Krachi West | 119 | 49417 | 2 | 0 | 3 | 0 | 0 | 15 | 7 | 0 | 38 | 20 | 14 | 7 |
| Northern | Kumbungu | 120 | 39341 | 116 | 13 | 10 | 17 | 39 | 23 | 6 | 11 | 15 | 43 | 67 | 13 |
| Ashanti | Kwabre East | 121 | 115556 | 33 | 8 | 28 | 23 | 39 | 15 | 5 | 19 | 29 | 33 | 38 | 34 |
| Eastern | Kwaebibirem | 122 | 113721 | 125 | 123 | 99 | 124 | 127 | 138 | 164 | 231 | 209 | 170 | 251 | 284 |
| Eastern | Kwahu North | 123 | 102423 | 17 | 17 | 9 | 32 | 34 | 39 | 20 | 27 | 20 | 21 | 24 | 22 |
| Eastern | Afram Plains South | 124 | 115812 | 8 | 14 | 3 | 8 | 23 | 8 | 0 | 1 | 10 | 48 | 12 | 9 |
| Eastern | Kwahu East | 125 | 77125 | 8 | 12 | 3 | 16 | 31 | 28 | 48 | 11 | 21 | 42 | 17 | 12 |
| Eastern | Kwahu South | 126 | 69757 | 32 | 35 | 38 | 24 | 16 | 20 | 38 | 30 | 47 | 26 | 19 | 24 |
| Eastern | Kwahu West | 127 | 93584 | 65 | 41 | 32 | 44 | 30 | 53 | 71 | 91 | 58 | 62 | 31 | 27 |
| Greater Accra | La-Dade-Kotopon | 128 | 183528 | 157 | 109 | 151 | 92 | 71 | 146 | 164 | 163 | 115 | 168 | 125 | 102 |
| Greater Accra | La-Nkwantanang-Madina | 129 | 111926 | 29 | 186 | 84 | 71 | 167 | 136 | 299 | 184 | 159 | 229 | 160 | 202 |
| Upper West | Lambussie-Karni | 130 | 51654 | 20 | 19 | 1 | 0 | 10 | 1 | 6 | 21 | 35 | 86 | 43 | 18 |
| Upper West | Lawra | 131 | 54889 | 2 | 1 | 1 | 2 | 6 | 4 | 3 | 0 | 3 | 7 | 6 | 0 |
| Greater Accra | Ledzokuku-Krowor | 132 | 227932 | 253 | 268 | 92 | 250 | 326 | 325 | 338 | 245 | 142 | 245 | 169 | 231 |
| Eastern | Lower-Manya Krobo | 133 | 89246 | 117 | 91 | 177 | 111 | 146 | 98 | 221 | 237 | 76 | 51 | 160 | 115 |
| Ashanti | Asante-Mampong | 134 | 88051 | 190 | 258 | 202 | 152 | 164 | 233 | 222 | 167 | 120 | 158 | 195 | 114 |
| Northern | Mamprugu-Moagduri | 135 | 46894 | 0 | 0 | 5 | 0 | 1 | 1 | 0 | 2 | 0 | 0 | 0 | 0 |
| Northern | East Mamprusi | 136 | 121009 | 63 | 39 | 51 | 105 | 34 | 59 | 7 | 65 | 19 | 56 | 16 | 20 |
| Central | Mfantsiman | 137 | 144332 | 149 | 245 | 116 | 71 | 143 | 163 | 182 | 280 | 142 | 131 | 143 | 116 |
| Northern | Mion | 138 | 81812 | 1 | 3 | 4 | 7 | 13 | 19 | 3 | 3 | 5 | 0 | 10 | 3 |
| Western | Mpohor | 139 | 42923 | 1 | 13 | 0 | 0 | 3 | 2 | 0 | 1 | 24 | 9 | 1 | 2 |
| Upper East | Nabdam | 140 | 33826 | 123 | 183 | 254 | 14 | 196 | 33 | 583 | 133 | 454 | 181 | 162 | 229 |
| Upper West | Nadowli-Kaleo | 141 | 61561 | 109 | 47 | 34 | 14 | 32 | 66 | 71 | 86 | 4 | 66 | 76 | 37 |
| Upper West | Nandom | 142 | 46040 | 36 | 52 | 55 | 59 | 66 | 79 | 99 | 118 | 86 | 83 | 98 | 44 |
| Northern | Nanumba North | 143 | 141584 | 12 | 7 | 6 | 5 | 8 | 6 | 7 | 14 | 8 | 13 | 19 | 49 |
| Northern | Nanumba South | 144 | 93464 | 0 | 24 | 0 | 0 | 0 | 0 | 0 | 0 | 0 | 0 | 0 | 0 |
| Eastern | New Juaben | 145 | 183727 | 254 | 346 | 314 | 276 | 264 | 464 | 444 | 498 | 347 | 315 | 274 | 323 |
| Greater Accra | Ningo Prampram | 146 | 70923 | 22 | 23 | 24 | 22 | 58 | 95 | 95 | 91 | 72 | 99 | 75 | 73 |
| Brong Ahafo | Nkoranza North | 147 | 65895 | 0 | 3 | 1 | 0 | 0 | 0 | 0 | 0 | 0 | 1 | 0 | 0 |
| Brong Ahafo | Nkoranza South | 148 | 100929 | 35 | 165 | 159 | 80 | 52 | 74 | 117 | 87 | 39 | 87 | 10 | 16 |
| Volta | Nkwanta North | 149 | 64553 | 56 | 55 | 67 | 101 | 149 | 107 | 126 | 142 | 181 | 143 | 98 | 99 |
| Volta | Nkwanta South | 150 | 117878 | 72 | 79 | 77 | 70 | 21 | 200 | 50 | 160 | 65 | 164 | 185 | 44 |
| Volta | North Dayi | 151 | 39913 | 13 | 0 | 1 | 12 | 36 | 0 | 2 | 11 | 2 | 2 | 1 | 0 |
| Northern | North Gonja | 152 | 43547 | 0 | 0 | 0 | 0 | 6 | 7 | 2 | 0 | 0 | 0 | 0 | 0 |
| Volta | North Tongu | 153 | 89777 | 27 | 38 | 30 | 19 | 110 | 85 | 77 | 41 | 0 | 0 | 0 | 0 |
| Eastern | Nsawam-Adoagyiri | 154 | 86000 | 93 | 93 | 78 | 49 | 89 | 105 | 100 | 164 | 64 | 53 | 77 | 88 |
| Western | Nzema East | 155 | 60828 | 20 | 22 | 0 | 26 | 13 | 13 | 38 | 22 | 33 | 35 | 11 | 20 |
| Ashanti | Obuasi | 156 | 168641 | 281 | 250 | 253 | 247 | 338 | 110 | 328 | 371 | 129 | 160 | 183 | 112 |
| Ashanti | Offinso | 157 | 76895 | 141 | 106 | 98 | 104 | 117 | 121 | 233 | 163 | 180 | 182 | 242 | 152 |
| Ashanti | Offinso North | 158 | 56881 | 81 | 27 | 223 | 162 | 138 | 226 | 205 | 115 | 78 | 100 | 66 | 93 |
| Western | Prestea-Huni Valley | 159 | 159304 | 61 | 43 | 93 | 20 | 62 | 31 | 38 | 25 | 44 | 25 | 14 | 8 |
| Brong Ahafo | Pru | 160 | 129248 | 159 | 272 | 171 | 24 | 129 | 45 | 65 | 58 | 64 | 58 | 32 | 23 |
| Upper East | Pusiga | 161 | 57677 | 1 | 72 | 0 | 101 | 93 | 115 | 45 | 71 | 50 | 89 | 110 | 119 |
| Northern | Saboba | 162 | 65706 | 24 | 63 | 98 | 64 | 57 | 74 | 372 | 274 | 192 | 291 | 158 | 149 |
| Northern | Sagnarigu | 163 | 148099 | 6 | 5 | 6 | 3 | 7 | 16 | 9 | 9 | 12 | 0 | 6 | 6 |
| Northern | Savelugu-Nanton | 164 | 139283 | 48 | 22 | 58 | 16 | 42 | 78 | 79 | 84 | 122 | 225 | 191 | 190 |
| Northern | Sawla-Tuna-Kalba | 165 | 99863 | 147 | 2 | 3 | 50 | 18 | 71 | 13 | 110 | 110 | 212 | 201 | 175 |
| Western | Sefwi-Akontombra | 166 | 82467 | 14 | 32 | 13 | 37 | 37 | 25 | 20 | 37 | 21 | 41 | 39 | 50 |
| Western | Bibiani-Anhwiaso-Bekwai | 167 | 123272 | 84 | 161 | 213 | 70 | 111 | 97 | 112 | 392 | 184 | 399 | 447 | 307 |
| Western | Sefwi-Wiawso | 168 | 139200 | 116 | 109 | 140 | 122 | 130 | 142 | 113 | 81 | 140 | 126 | 159 | 115 |
| Western | Sekondi-Takoradi | 169 | 559548 | 55 | 60 | 81 | 84 | 104 | 124 | 248 | 31 | 98 | 117 | 102 | 90 |
| Ashanti | Sekyere-Afram Plains | 170 | 65402 | 1 | 2 | 0 | 2 | 0 | 0 | 0 | 0 | 0 | 0 | 1 | 0 |
| Ashanti | Sekyere-Kumawu | 171 | 28535 | 8 | 5 | 2 | 1 | 2 | 6 | 3 | 8 | 5 | 5 | 1 | 0 |
| Ashanti | Sekyere Central | 172 | 71232 | 24 | 11 | 30 | 31 | 26 | 21 | 11 | 24 | 18 | 25 | 20 | 9 |
| Ashanti | Sekyere East | 173 | 62172 | 60 | 14 | 27 | 51 | 57 | 45 | 63 | 124 | 58 | 94 | 80 | 67 |
| Brong Ahafo | Sene East | 174 | 61076 | 10 | 11 | 0 | 0 | 0 | 8 | 11 | 13 | 19 | 18 | 16 | 17 |
| Brong Ahafo | Sene West | 175 | 57734 | 19 | 12 | 0 | 2 | 1 | 0 | 4 | 2 | 23 | 5 | 4 | 5 |
| Greater Accra | Shai-Osudoku | 176 | 51913 | 8 | 11 | 9 | 10 | 21 | 21 | 45 | 38 | 18 | 43 | 32 | 43 |
| Western | Shama | 177 | 81966 | 59 | 24 | 32 | 14 | 27 | 44 | 35 | 38 | 40 | 22 | 27 | 37 |
| Upper West | Sissala East | 178 | 56528 | 18 | 1 | 0 | 0 | 11 | 2 | 3 | 0 | 0 | 1 | 6 | 7 |
| Upper West | Sissala West | 179 | 49573 | 7 | 55 | 20 | 33 | 25 | 34 | 22 | 33 | 11 | 36 | 32 | 34 |
| Volta | South Dayi | 180 | 46661 | 24 | 41 | 18 | 0 | 9 | 19 | 33 | 22 | 10 | 19 | 1 | 2 |
| Volta | South Tongu | 181 | 87950 | 50 | 3 | 5 | 0 | 10 | 29 | 42 | 25 | 22 | 0 | 0 | 8 |
| Western | Suaman | 182 | 20529 | 7 | 6 | 30 | 9 | 17 | 8 | 17 | 19 | 7 | 6 | 8 | 5 |
| Eastern | Suhum | 183 | 90358 | 163 | 274 | 248 | 352 | 172 | 168 | 244 | 237 | 110 | 244 | 263 | 90 |
| Brong Ahafo | Sunyani Municipal | 184 | 123224 | 31 | 73 | 42 | 94 | 127 | 111 | 79 | 58 | 154 | 115 | 143 | 203 |
| Brong Ahafo | Sunyani West | 185 | 85272 | 14 | 36 | 33 | 67 | 27 | 41 | 43 | 23 | 13 | 16 | 20 | 35 |
| Brong Ahafo | Tain | 186 | 88104 | 15 | 24 | 26 | 23 | 17 | 7 | 2 | 5 | 0 | 2 | 5 | 6 |
| Upper East | Talensi | 187 | 81194 | 12 | 41 | 87 | 107 | 64 | 100 | 62 | 29 | 76 | 46 | 44 | 42 |
| Northern | Tamale | 188 | 223252 | 127 | 208 | 13 | 4 | 39 | 42 | 34 | 74 | 42 | 51 | 118 | 102 |
| Brong Ahafo | Tano North | 189 | 79973 | 29 | 10 | 11 | 16 | 17 | 12 | 9 | 30 | 27 | 34 | 26 | 16 |
| Brong Ahafo | Tano South | 190 | 78129 | 13 | 13 | 15 | 7 | 10 | 12 | 14 | 15 | 17 | 19 | 21 | 23 |
| Western | Tarkwa-Nsuaem | 191 | 90477 | 625 | 524 | 350 | 688 | 397 | 764 | 500 | 515 | 390 | 558 | 467 | 385 |
| Northern | Tatale-Sangule | 192 | 60039 | 21 | 18 | 33 | 18 | 57 | 38 | 8 | 33 | 0 | 16 | 30 | 13 |
| Brong Ahafo | Techiman Municipal | 193 | 147788 | 83 | 2 | 53 | 87 | 92 | 116 | 250 | 48 | 51 | 106 | 223 | 213 |
| Brong Ahafo | Techiman North | 194 | 59068 | 0 | 0 | 0 | 0 | 0 | 0 | 0 | 0 | 0 | 0 | 0 | 0 |
| Greater Accra | Tema | 195 | 292773 | 139 | 195 | 200 | 117 | 203 | 243 | 280 | 156 | 99 | 161 | 89 | 99 |
| Northern | Tolon | 196 | 72990 | 8 | 12 | 12 | 14 | 25 | 23 | 13 | 17 | 5 | 30 | 11 | 2 |
| Central | Twifo-Ati-Mokwa | 197 | 61743 | 0 | 1 | 0 | 1 | 0 | 4 | 0 | 6 | 6 | 1 | 2 | 0 |
| Central | Twifo-Hemang Lower Denkyira | 198 | 55131 | 15 | 44 | 25 | 12 | 11 | 13 | 26 | 19 | 8 | 19 | 16 | 10 |
| Central | Upper Denkyira East | 199 | 72810 | 101 | 128 | 98 | 146 | 244 | 89 | 82 | 68 | 47 | 39 | 39 | 192 |
| Central | Upper Denkyira West | 200 | 60054 | 185 | 161 | 155 | 40 | 105 | 214 | 110 | 113 | 161 | 194 | 95 | 193 |
| Eastern | Upper Manya-Krobo | 201 | 72092 | 26 | 26 | 25 | 25 | 15 | 18 | 9 | 37 | 18 | 10 | 12 | 28 |
| Eastern | Upper West Akim | 202 | 87051 | 1 | 12 | 8 | 0 | 8 | 13 | 15 | 46 | 36 | 36 | 43 | 23 |
| Upper West | Wa East | 203 | 72074 | 0 | 4 | 14 | 2 | 0 | 22 | 15 | 2 | 2 | 11 | 8 | 1 |
| Upper West | Wa | 204 | 107214 | 20 | 88 | 78 | 63 | 62 | 77 | 59 | 100 | 48 | 192 | 213 | 98 |
| Upper West | Wa West | 205 | 81348 | 1 | 6 | 22 | 0 | 7 | 10 | 3 | 0 | 8 | 0 | 2 | 0 |
| Western | Wassa-Amenfi Central | 206 | 69014 | 10 | 29 | 20 | 32 | 5 | 26 | 27 | 21 | 0 | 8 | 7 | 7 |
| Western | Wassa Amenfi East | 207 | 83478 | 0 | 8 | 11 | 20 | 31 | 69 | 44 | 44 | 18 | 0 | 34 | 10 |
| Western | Wassa Amenfi West | 208 | 92152 | 139 | 98 | 147 | 140 | 148 | 111 | 188 | 211 | 55 | 151 | 186 | 126 |
| Western | Wassa East | 209 | 81073 | 17 | 25 | 39 | 62 | 37 | 107 | 51 | 45 | 33 | 41 | 35 | 17 |
| Brong Ahafo | Wenchi | 210 | 89739 | 8 | 1 | 11 | 0 | 2 | 0 | 3 | 1 | 0 | 0 | 0 | 0 |
| Eastern | West Akim | 211 | 108298 | 129 | 135 | 218 | 201 | 220 | 240 | 232 | 254 | 281 | 303 | 298 | 387 |
| Northern | West Gonja | 212 | 41180 | 76 | 54 | 109 | 110 | 139 | 162 | 160 | 137 | 169 | 85 | 143 | 180 |
| Northern | West Mamprusi | 213 | 121117 | 0 | 31 | 42 | 5 | 26 | 26 | 10 | 11 | 13 | 33 | 13 | 32 |
| Northern | Yendi | 214 | 117780 | 88 | 109 | 59 | 88 | 80 | 135 | 229 | 212 | 277 | 330 | 257 | 189 |
| Eastern | Yilo-Krobo | 215 | 87847 | 14 | 10 | 2 | 21 | 4 | 0 | 24 | 0 | 11 | 7 | 12 | 6 |
| Northern | Zabzugu | 216 | 63815 | 110 | 106 | 87 | 51 | 103 | 70 | 106 | 67 | 44 | 85 | 79 | 48 |
